# Evaluating inter-rater reliability in the context of “Sysmex UN2000 detection of protein/creatinine ratio and of renal tubular epithelial cells can be used for screening lupus nephritis”: a statistical examination

**DOI:** 10.1186/s12882-024-03540-y

**Published:** 2024-03-13

**Authors:** Ming Li, Qian Gao, Jing Yang, Tianfei Yu

**Affiliations:** 1 Department of Software Engineering, College of Computer Science and Technology, Harbin Engineering University, 150001 Harbin, China; 2 Department of Computer Science and Technology, College of Computer and Control Engineering, Qiqihar University, 161006 Qiqihar, China; 3 Heilongjiang Provincial Key Laboratory of Resistance Gene Engineering and Protection of Bioaffiliationersity in Cold Areas, Qiqihar University, 161006 Qiqihar, China; 4 Department of Biotechnology, College of Life Science and Agriculture Forestry, Qiqihar University, 161006 Qiqihar, China

**Keywords:** Lupus nephritis, AU5800, UN2000, Inter-rater reliability, Kappa statistic, Cohen’s Kappa, Weighted Kappa

## Abstract

**Background:**

The evaluation of inter-rater reliability (IRR) is integral to research designs involving the assessment of observational ratings by two raters. However, existing literature is often heterogeneous in reporting statistical procedures and the evaluation of IRR, although such information can impact subsequent hypothesis testing analyses.

**Methods:**

This paper evaluates a recent publication by Chen et al., featured in *BMC Nephrology*, aiming to introduce an alternative statistical approach to assessing IRR and discuss its statistical properties. The study underscores the crucial need for selecting appropriate Kappa statistics, emphasizing the accurate computation, interpretation, and reporting of commonly used IRR statistics between two raters.

**Results:**

The Cohen’s Kappa statistic is typically used for two raters dealing with two categories or for unordered categorical variables having three or more categories. On the other hand, when assessing the concordance between two raters for ordered categorical variables with three or more categories, the commonly employed measure is the weighted Kappa.

**Conclusion:**

Chen and colleagues might have underestimated the agreement between AU5800 and UN2000. Although the statistical approach adopted in Chen et al.’s research did not alter their findings, it is important to underscore the importance of researchers being discerning in their choice of statistical techniques to address their specific research inquiries.

The evaluation of inter-rater reliability (IRR) is integral to research designs involving the assessment of observational ratings by two raters. However, existing literature is often heterogeneous in reporting statistical procedures and the evaluation of IRR, although such information can impact subsequent hypothesis testing analyses [1, 2]. This commentary evaluates a recent publication by Chen et al. [3], featured in *BMC Nephrology*, aiming to introduce an alternative statistical approach to assessing IRR and discuss its statistical properties. The study underscores the crucial need for selecting appropriate Kappa statistics, emphasizing the accurate computation, interpretation, and reporting of commonly used IRR statistics between two raters. The analysis focuses on the raised issues, contributing to the discourse on IRR assessment methodology. We are committed to addressing these concerns comprehensively, providing insights into the selection of suitable statistical measures for robust IRR evaluation. This effort aims to elevate the quality of research practices in IRR assessment, thereby fostering a more accurate and reliable foundation for scientific investigations across diverse fields.

## Kappa statistic

When delving into the examination of nominal and categorical data, researchers commonly turn to classical statistical techniques such as the Kappa statistic and its iterations like Cohen’s Kappa. These metrics are instrumental in gauging the agreement among diverse observers, a pivotal aspect for ensuring methodological rigor and reliability in research. Through their application, researchers aim to precisely evaluate the consistency of ratings, establishing a robust foundation for the integrity of their findings and the overall validity of their study.

### Cohen’s Kappa

Cohen’s Kappa, a widely utilized statistical method for assessing IRR, presents certain limitations [4]. Primarily designed for fully-crossed designs with precisely two raters, Cohen’s Kappa may exhibit biases and limitations in certain scenarios. Unlike simple percent agreement, it accounts for chance agreement, emphasizing its utility for two raters dealing with two categories or for unordered categorical variables having three or more categories [5–7]. Recognizing these constraints is crucial when employing Cohen’s Kappa, urging researchers to consider alternative approaches tailored to their specific study designs and categorical data structures.

Cohen’s Kappa is calculated as follows:

1 $$ {k}_{C}=\frac{\sum _{j=1}^{n}{u}_{jj}\left(i{i}^{{\prime }}\right)-\sum _{j=1}^{n}{p}_{ij}{p}_{{i}^{{\prime }}j}}{1-\sum _{j=1}^{n}{p}_{ij}{p}_{{i}^{{\prime }}j}}$$

The value of $$ {u}_{jj}\left(i{i}^{{\prime }}\right)$$ is the proportion of objects put in the same category *j* by both raters $$ i$$ and $$ {i}^{{\prime }}$$. The value of $$ {p}_{ij}$$ is the proportion of objects that rater $$ i$$ assigned to category $$ j$$.

## Weighted Kappa

When assessing the concordance between two raters for ordered categorical variables with three or more categories, the commonly employed measure is the weighted Kappa [8–10]. Two variations of weighted Kappa exist: the linear weighted Kappa (LWK) [11] and the quadratic weighted Kappa (QWK) [12]. LWK assigns weights based on linear distances between categories, while QWK uses quadratic distances. Both LWK and QWK offer more nuanced insights into IRR compared to Cohen’s Kappa. The selection between LWK and QWK hinges on the nature of the data. To ensure a comprehensive understanding of disagreements, it is advisable to report both coefficients, particularly in situations where not all disagreements hold equal significance [13]. This dual reporting strategy contributes to a more thorough evaluation of the distribution of disagreements [14], enhancing the accuracy and depth of the assessment of consistency and reliability in intricate datasets.

Weighted Kappa is calculated as follows:

2 $$ {w}_{ij}^{\left(m\right)}=1-{\left(\left.\frac{\left|i-j\right|}{n-1}\right)\right.}^{m}$$

3 $$ {k}_{m}=1-\frac{1-\sum _{i=1}^{n}\sum _{j=1}^{n}{w}_{ij}^{\left(m\right)}{p}_{ij}}{1-\sum _{i=1}^{n}\sum _{j=1}^{n}{w}_{ij}^{\left(m\right)}{p}_{i}{q}_{j}}$$

Where m ≥ 1, $$ p$$ and $$ q$$ are relative frequencies, which reflect the proportion of frequency to the number of samples. $$ {p}_{i}={\sum }_{j=1}^{n}{p}_{ij} $$ and $$ {q}_{i}={\sum }_{j=1}^{n}{p}_{ji}$$. In special cases, $$ {k}_{1}$$ is the LWK and $$ {k}_{2}$$ is the QWK.

## The interpretation of Kappa value

Cohen devised a classification system for interpreting Kappa values, indicating various levels of agreement [4]. However, McHugh [15] highlighted practical concerns, arguing that labeling a 61% agreement rate as “substantial” might be misleading, especially in critical settings like clinical laboratories where a 40% error rate would be significant. He emphasized the need for a higher standard, with many sources recommending a minimum interrater agreement of 80%. McHugh proposed an alternative interpretation of Kappa values, categorizing ≤ 0.20 as no agreement, 0.21 to 0.39 as minimal agreement, 0.40 to 0.59 as weak agreement, 0.60 to 0.79 as moderate agreement, 0.80 to 0.90 as strong agreement, and values exceeding 0.90 as almost perfect agreement. This alternative approach considers the practical implications of different Kappa values, offering a nuanced perspective on agreement levels in situations where accuracy has substantial real-world consequences. By addressing these concerns, McHugh’s interpretation provides a more contextually relevant framework for understanding and applying Kappa values, particularly in critical decision-making environments.

### Comparative Kappa statistics between Cohen’s and weighted approaches

Chen et al. assessed the viability of utilizing automated urine sediment analysis (UN2000) for lupus nephritis screening. 284 urine samples from systemic lupus erythematosus patients were examined with UN2000, evaluating protein/creatinine ratio (P/C) and renal tubular epithelial cells. Employing biochemical analysis and microscopy as the gold standard, the Kappa consistency test demonstrated strong and good agreement for P/C and renal tubular epithelial cells (RTEC), respectively (Cohen’s Kappa, 0.858). Setting P/C ≥ 2 + as the sole screening standard yielded the highest specificity, positive predictive value, and coincidence for lupus nephritis. Combining P/C ≥ 2 + or RTEC > 2.8 cells/µl as the standard maximized sensitivity and negative predictive value. UN2000 proves effective in lupus nephritis screening by detecting P/C and RTEC. Yet, as mentioned earlier, in the context of three-category ordinal variables, opting for weighted Kappa is often a more suitable approach for evaluating IRR compared to Cohen’s Kappa.

Upon examining the data provided by the authors, the agreement between AU5800 and UN2000 was assessed using three Kappa values with SPSSAU (https://spssau.com/) (Table 1). There was strong agreement between the two categories, with Cohen’s Kappa, and almost complete agreement with LWK and QWK. As a result, LWK and QWK are the preferred measures for more sensitive evaluation that emphasizes larger differences in judgment when assessing agreement.

**Table 1:** The Kappa coefficient between the AU5800 and UN2000

Test parameters		UN2000*						
		-	1+	2+	Total	κ_c_	κ_lW_	κ_qW_
AU5800(mg/g)	< 150	427	21	0	448	0.858 (*p* < 0.001, 95% CI = 0.816–0.899)	0.909 (*p* < 0.001, 95% CI = 0.882–0.937)	0.947 (*p* < 0.001, 95% CI = 0.931–0.964)
	150 ~ 490	4	48	8	60			
	≧ 500	0	7	105	112			
	Total	431	76	113	620			

The data has been cited from the article published by Chen et al. [3]. κ_c_: Cohen’s Kappa value; κ_lw_: linear weighted Kappa value; κ_qw_: quadratic weighted Kappa value; CI: confidence interval.

## Conclusion

In conclusion, Chen and colleagues might have underestimated the agreement between AU5800 and UN2000. When choosing an IRR statistical test, researchers should consider variable coding, study design, and the purpose of the estimate. It’s crucial to assess the statistic’s suitability and explore alternatives. Although the statistical approach adopted in Chen et al.’s research did not alter their findings, it is important to underscore the importance of researchers being discerning in their choice of statistical techniques to address their specific research inquiries.

## Data Availability

Data sharing not applicable to this article as no datasets were generated or analyzed during the current study.

## Abbreviations

IRR: inter rater reliability
LWK: linear weighted Kappa
QWK: quadratic weighted Kappa
P/C: protein/creatinine ratio
RTEC: renal tubular epithelial cells
κ_c_: Cohen’s Kappa value
κ_lw_: linear weighted Kappa Value
κ_qw_: quadratic weighted Kappa value
CI: confidence interval
